# Tris[(1,4,7,10,13,16-hexa­oxacyclo­octa­deca­ne)rubidium] heptaantimonide–ammonia (1/4)

**DOI:** 10.1107/S1600536811041237

**Published:** 2011-10-22

**Authors:** Fabian Mutzbauer, Nikolaus Korber

**Affiliations:** a Institut für Anorganische Chemie, Universität Regensburg, Universitätsstrasse 31, 93053 Regensburg, Germany

## Abstract

The crystal structure of the title compound, [Rb(C_12_H_24_O_6_)]_3_[Sb_7_]·4NH_3_, fills the gap between the already known Zintl anion ammoniates {[Cs(18-crown-6)]_3_Sb_7_}_2_·9NH_3_ [Wiesler (2007 ▶). Dissertation, Universität Regensburg, Germany] and [K(18-crown-6)]_3_Sb_7_·4NH_3_ [Hanauer (2007 ▶). Dissertation, Universität Regensburg, Germany]. As in the two known compounds, the anti­mony cage anion in this crystal structure is coordinated by three alkali cations. The coordination spheres of each of the cations are saturated by 18-crown-6 mol­ecules. The ammonia mol­ecules of crystallization are situated between the crown ethers. The neutral, mol­ecular [Rb(18-crown-6)]_3_Sb_7_ units are inter­connected by multiple dipole–dipole interactions between ammonia and 18-crown-6.

## Related literature

Rb_3_Sb_7_ can be obtained by a high-temperature solid-state reaction (Hirschle & Röhr, 2000*a* ▶) like the homologous Cs_3_Sb_7_ phase (Hirschle & Röhr, 2000*b* ▶). By dissolving these solids in solvents like ethyl­enediamine or liquid ammonia in the presence of chelating ligands like crown ether or cryptand mol­ecules, new solvent-rich compounds can be crystallized from the mother liquor, see: Critchlow & Corbett (1984 ▶); Adolphson *et al.* (1976 ▶); Kummer *et al.* (1976 ▶); Hanauer (2007 ▶); Wiesler (2007 ▶). For the isotypic structure [K(18-crown-6)]_3_Sb_7_·4NH_3_, see: Hanauer (2007 ▶). For the specification of nortricyclane analogue cluster anions, see: Hönle & von Schnering (1978 ▶); Somer *et al.* (1989 ▶). 
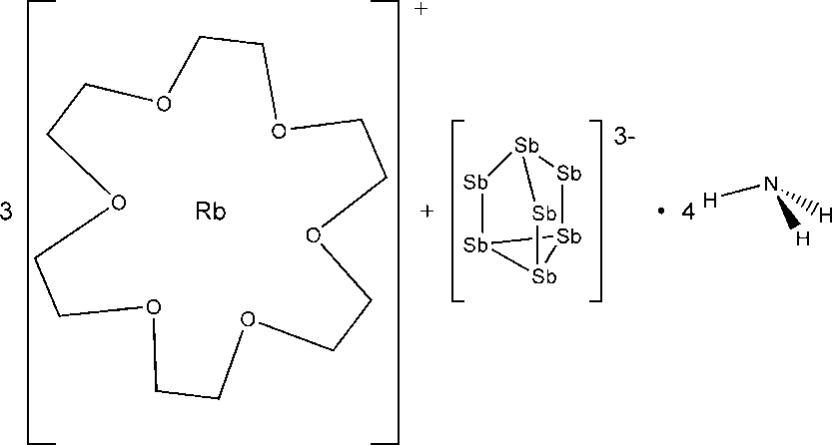


## Experimental

### 

#### Crystal data

[Rb(C_12_H_24_O_6_)]_3_[Sb_7_]·4NH_3_
*M* *_r_* = 1969.73
Monoclinic, 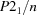

*a* = 15.000 (3) Å
*b* = 17.484 (4) Å
*c* = 25.158 (5) Å
β = 90.98 (3)°
*V* = 6597 (2) Å^3^
*Z* = 4
Mo *K*α radiation
μ = 5.08 mm^−1^
*T* = 123 K
0.3 × 0.2 × 0.1 mm

#### Data collection

Stoe IPDS1 diffractometer
Absorption correction: numerical (*X-RED*/*X-SHAPE* in *X-AREA*; Stoe & Cie, 2005 ▶) *T*_min_ = 0.453, *T*_max_ = 0.648
88182 measured reflections
12127 independent reflections
9417 reflections with *I* > 2σ(*I*)
*R*_int_ = 0.090

#### Refinement

*R*[*F* ^2^ > 2σ(*F* ^2^)] = 0.036
*wR*(*F* ^2^) = 0.083
*S* = 0.96
12127 reflections
617 parameters
H-atom parameters constrained
Δρ_max_ = 1.66 e Å^−3^
Δρ_min_ = −0.74 e Å^−3^

### 

Data collection: *X-AREA* (Stoe & Cie, 2005 ▶); cell refinement: *X-AREA*; data reduction: *X-AREA*; program(s) used to solve structure: *SHELXS97* (Sheldrick, 2008 ▶); program(s) used to refine structure: *SHELXL97* (Sheldrick, 2008 ▶); molecular graphics: *DIAMOND* (Brandenburg, 2001 ▶); software used to prepare material for publication: *publCIF* (Westrip, 2010 ▶).

## Supplementary Material

Crystal structure: contains datablock(s) I, global. DOI: 10.1107/S1600536811041237/hp2013sup1.cif

Structure factors: contains datablock(s) I. DOI: 10.1107/S1600536811041237/hp2013Isup2.hkl

Additional supplementary materials:  crystallographic information; 3D view; checkCIF report

## Figures and Tables

**Fig. 1.**
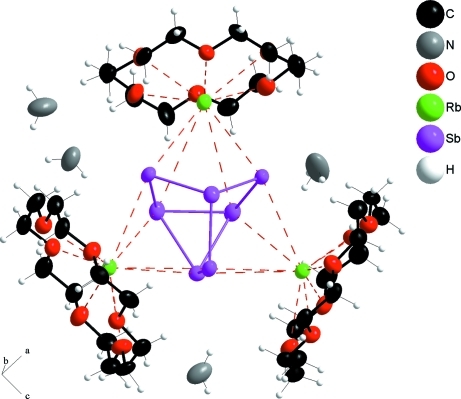
: Asymmetric unit of the compound [Rb(18-crown-6)]3Sb7.4NH3. Ellipsoids of all non-hydrogen atoms are given with a probability factor of 70%.

**Fig. 2.**
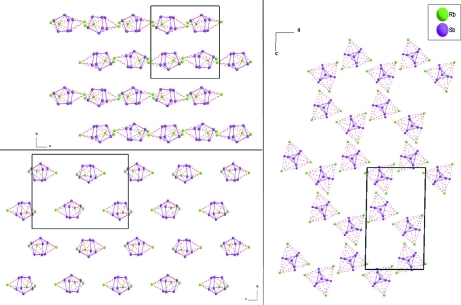
: Packing of the [Rb(18-crown-6)]3Sb7 units in each crystallographic direction. Crown ethers and ammonia molecules are omitted. The probability factor of the mapped atoms is 70%.

## supplementary crystallographic information

### Comment

The compound Rb_3_Sb_7_ can be obtained by a high temperature solid state reaction (Hirschle & Röhr, 2000*a*) like the homologous Cs_3_Sb_7_ phase (Hirschle & Röhr, 2000*b*). By dissolving these solids in solvents like ethylenediamine or liquid ammonia in the presence of chelating ligands like crown ether or cryptand molecules, new solvent rich compounds can be crystallized from the mother liquor (Critchlow & Corbett, 1984; Adolphson *et al.*,  1976; Kummer *et al.*, 1976; Hanauer, 2007; Wiesler, 2007). There is a line of crystal structures documented showing a distinct progression from the pure solid crystal to a solvent rich crystal. In the pure solid phase, the anion is coordinated directly by cations. The solvent rich crystal structures contain cations which are coordinated by chelating ligands and/or solvent molecules. This yields anionic cluster molecules which only feature weak ion-dipole interactions. The here presented [Rb(18-crown-6)]_3_Sb_7_.4NH_3_ compound is isostructural to the crystal structure of [K(18-crown-6)]_3_Sb_7_.4NH_3_ (Hanauer, 2007). Each rubidium cation binds exclusively to one crytallographically independent Sb_7_ cage in an η^4^-like fashion. To complete a coordination number of ten for each metal atom, it is saturated by one 18-crown-6 molecule (Fig. 1). Four ammonia molecules are localized between the three crown ether ligands of each unit. These solvent molecules interact by hydrogen bonding with crown ether molecules and ammonia molecules of adjacent [Rb(18-crown-6)]_3_Sb_7_ × 4NH_3_ units. Therefore, the structure can be described as a packing of isolated [Rb(18-crown-6)]_3_Sb_7_ units. This packing and the orientation of these units is shown in Figure 2. The nortricyclane analogue cluster anions were specified by von Schnering *et al.* They defined the cluster by its height *H* and the quotient *Q* between *H* and the average of the three bonding distances between the three atoms of the triangular base area (Hönle & von Schnering, 1978; Somer *et al.*, 1989). The presented Sb_7_ anion shows characteristic values for this kind of cage of *H* = 3.8653 (5) Å and *Q* = 1.33.

### Experimental

All preparations were carried out in an atmosphere of dryed argon (99.9996%). 173 mg Rb_3_Sb_7_ (0.156 mmol), 41 mg 18-crown-6 (0.156 mmol) and 100 mg [Ni(CO)_2_(PPh_3_)_2_] (0.156 mmol) were placed in a baked out reaction vessel inside a glove box. Afterwards ammonia (99.99990%) was condensed onto the solids until a filling level of about 15 ml solvent was achieved. A light brown suspension resulted. After 3 month of storage at 233 K a dark brown solution could be obtained and dark brown crystals could be isolated.

### Refinement

The hydrogen atoms of the crown ether and the ammonia molecules were generated using the HFIX instruction.

### Crystal data

**Table:**

[Rb(C_12_H_24_O_6_)]_3_[Sb_7_]·4NH_3_	*F*(000) = 3760
*M**_r_* = 1969.73	*D*_x_ = 1.983 Mg m^−^^3^
Monoclinic, *P*2_1_/*n*	Mo *K*α radiation, λ = 0.71073 Å
Hall symbol: -P 2yn	Cell parameters from 12127 reflections
*a* = 15.000 (3) Å	θ = 2.0–25.5°
*b* = 17.484 (4) Å	µ = 5.08 mm^−^^1^
*c* = 25.158 (5) Å	*T* = 123 K
β = 90.98 (3)°	Block, clear brown
*V* = 6597 (2) Å^3^	0.3 × 0.2 × 0.1 mm
*Z* = 4	

### Data collection

**Table:**

Stoe IPDS1 diffractometer	12127 independent reflections
Radiation source: fine-focus sealed tube	9417 reflections with *I* > 2σ(*I*)
graphite	*R*_int_ = 0.090
rotation scans	θ_max_ = 25.8°, θ_min_ = 2.0°
Absorption correction: numerical (*X-SHAPE* in *X-AREA*; Stoe & Cie, 2005)	*h* = −18→18
*T*_min_ = 0.453, *T*_max_ = 0.648	*k* = −21→21
88182 measured reflections	*l* = −30→30

### Refinement

**Table:**

Refinement on *F*^2^	Primary atom site location: structure-invariant direct methods
Least-squares matrix: full	Secondary atom site location: difference Fourier map
*R*[*F*^2^ > 2σ(*F*^2^)] = 0.036	Hydrogen site location: inferred from neighbouring sites
*wR*(*F*^2^) = 0.083	H-atom parameters constrained
*S* = 0.96	*w* = 1/[σ^2^(*F*_o_^2^) + (0.0446*P*)^2^] where *P* = (*F*_o_^2^ + 2*F*_c_^2^)/3
12127 reflections	(Δ/σ)_max_ = 0.005
617 parameters	Δρ_max_ = 1.66 e Å^−^^3^
0 restraints	Δρ_min_ = −0.74 e Å^−^^3^

### Special details

**Table:**

Experimental. crystal mounting in perfluorether (T. Kottke, D. Stalke, J. Appl. Crystallogr. 26, 1993, p. 615), tube power 1.65 kW, collimator size 0.5 mm, detector distance 70 mm, exposure time 600 s, phi increment 0.9°, phi range 0–360°, 2θ range 3.3–52.1°, d(*hkl*) range 0.809–12.453 Å
Geometry. All e.s.d.'s (except the e.s.d. in the dihedral angle between two l.s. planes) are estimated using the full covariance matrix. The cell e.s.d.'s are taken into account individually in the estimation of e.s.d.'s in distances, angles and torsion angles; correlations between e.s.d.'s in cell parameters are only used when they are defined by crystal symmetry. An approximate (isotropic) treatment of cell e.s.d.'s is used for estimating e.s.d.'s involving l.s. planes.
Refinement. Refinement of *F*^2^ against ALL reflections. The weighted *R*-factor *wR* and goodness of fit *S* are based on *F*^2^, conventional *R*-factors *R* are based on *F*, with *F* set to zero for negative *F*^2^. The threshold expression of *F*^2^ > σ(*F*^2^) is used only for calculating *R*-factors(gt) *etc*. and is not relevant to the choice of reflections for refinement. *R*-factors based on *F*^2^ are statistically about twice as large as those based on *F*, and *R*- factors based on ALL data will be even larger.

### Fractional atomic coordinates and isotropic or equivalent isotropic displacement parameters (Å^2^)

**Table:**

	*x*	*y*	*z*	*U*_iso_*/*U*_eq_	
Sb1	0.91679 (3)	0.20198 (3)	0.387503 (15)	0.02665 (9)	
Sb2	0.66946 (3)	0.33966 (2)	0.362555 (16)	0.02828 (10)	
Sb3	0.75841 (3)	0.12411 (2)	0.410855 (16)	0.02666 (9)	
Sb4	0.70480 (3)	0.19402 (3)	0.506203 (15)	0.02748 (10)	
Sb5	0.71263 (3)	0.34468 (2)	0.474027 (16)	0.02924 (10)	
Sb6	0.64158 (3)	0.18833 (3)	0.337947 (15)	0.02665 (9)	
Sb7	0.85396 (3)	0.35016 (2)	0.395167 (16)	0.02880 (10)	
Rb1	0.46710 (4)	0.21983 (3)	0.44607 (2)	0.02518 (12)	
Rb2	0.95927 (4)	0.22994 (3)	0.53318 (2)	0.02658 (12)	
Rb3	0.83514 (4)	0.24754 (3)	0.24583 (2)	0.02490 (12)	
O1	0.4008 (3)	0.1621 (3)	0.55500 (16)	0.0305 (9)	
O2	0.4390 (3)	0.3207 (3)	0.53895 (16)	0.0319 (10)	
O3	0.3847 (3)	0.3825 (3)	0.44145 (17)	0.0322 (10)	
O4	0.3449 (3)	0.1260 (3)	0.36889 (17)	0.0309 (9)	
O5	0.3690 (3)	0.2854 (3)	0.35223 (17)	0.0333 (10)	
O6	0.4027 (3)	0.0608 (3)	0.46431 (17)	0.0312 (10)	
O7	0.8969 (3)	0.1597 (3)	0.64160 (16)	0.0297 (9)	
O8	1.1401 (3)	0.1445 (3)	0.51457 (16)	0.0285 (9)	
O9	1.1337 (3)	0.3047 (3)	0.49512 (18)	0.0335 (10)	
O10	0.8821 (3)	0.3195 (3)	0.62418 (16)	0.0335 (10)	
O11	0.9924 (3)	0.0684 (2)	0.56761 (16)	0.0282 (9)	
O12	1.0241 (3)	0.3883 (3)	0.56777 (17)	0.0342 (10)	
O13	1.0195 (3)	0.3198 (3)	0.23963 (17)	0.0330 (10)	
O14	0.7086 (3)	0.1838 (2)	0.15877 (16)	0.0280 (9)	
O15	0.7067 (3)	0.3442 (2)	0.18593 (16)	0.0290 (9)	
O16	1.0121 (3)	0.1585 (3)	0.21902 (18)	0.0346 (10)	
O17	0.8777 (3)	0.4112 (3)	0.20401 (16)	0.0305 (9)	
O18	0.8425 (3)	0.0911 (2)	0.20396 (18)	0.0315 (10)	
N1	0.4990 (5)	0.3665 (4)	0.2515 (2)	0.0469 (16)	
H1C	0.5459	0.3338	0.2490	0.070*	
H1D	0.4724	0.3721	0.2189	0.070*	
H1E	0.4588	0.3474	0.2747	0.070*	
N2	0.6607 (5)	0.3774 (4)	0.6247 (2)	0.0504 (16)	
H2C	0.6689	0.4289	0.6269	0.076*	
H2D	0.6134	0.3636	0.6447	0.076*	
H2E	0.6501	0.3640	0.5902	0.076*	
N3	1.1030 (5)	0.3669 (6)	0.3577 (3)	0.068 (2)	
H3C	1.0855	0.3494	0.3899	0.102*	
H3D	1.0795	0.3366	0.3316	0.102*	
H3E	1.0834	0.4158	0.3530	0.102*	
N4	0.7473 (6)	0.4847 (5)	0.7235 (3)	0.070 (2)	
H4C	0.6910	0.4797	0.7099	0.105*	
H4D	0.7602	0.5352	0.7278	0.105*	
H4E	0.7510	0.4607	0.7556	0.105*	
C1	0.3557 (4)	0.0185 (4)	0.4250 (3)	0.0319 (14)	
H1A	0.2908	0.0224	0.4308	0.038*	
H1B	0.3728	−0.0361	0.4271	0.038*	
C2	0.9556 (4)	0.0390 (4)	0.6156 (2)	0.0318 (14)	
H2A	1.0001	0.0435	0.6449	0.038*	
H2B	0.9410	−0.0158	0.6109	0.038*	
C3	0.7783 (4)	0.0619 (4)	0.1680 (2)	0.0294 (13)	
H3A	0.7679	0.0070	0.1751	0.035*	
H3B	0.7991	0.0674	0.1311	0.035*	
C4	1.0808 (4)	0.1947 (4)	0.2498 (3)	0.0343 (14)	
H4A	1.1366	0.1647	0.2476	0.041*	
H4B	1.0633	0.1972	0.2875	0.041*	
C5	0.6926 (4)	0.1067 (4)	0.1752 (3)	0.0292 (13)	
H5A	0.6439	0.0839	0.1535	0.035*	
H5B	0.6750	0.1056	0.2130	0.035*	
C6	0.8218 (4)	0.2046 (4)	0.6591 (2)	0.0332 (14)	
H6A	0.7955	0.1808	0.6909	0.040*	
H6B	0.7754	0.2066	0.6306	0.040*	
C7	0.3157 (5)	0.2378 (4)	0.3193 (2)	0.0374 (16)	
H7A	0.3121	0.2596	0.2830	0.045*	
H7B	0.2546	0.2352	0.3334	0.045*	
C8	1.0744 (4)	0.0331 (4)	0.5527 (3)	0.0300 (14)	
H8A	1.0641	−0.0221	0.5464	0.036*	
H8B	1.1186	0.0383	0.5821	0.036*	
C9	0.8724 (4)	0.0825 (4)	0.6298 (2)	0.0318 (14)	
H9A	0.8291	0.0812	0.5997	0.038*	
H9B	0.8442	0.0587	0.6610	0.038*	
C10	1.2111 (4)	0.2592 (4)	0.4849 (3)	0.0339 (14)	
H10A	1.2498	0.2571	0.5172	0.041*	
H10B	1.2456	0.2824	0.4559	0.041*	
C11	0.7288 (4)	0.4210 (4)	0.1731 (3)	0.0321 (14)	
H11A	0.6744	0.4530	0.1733	0.039*	
H11B	0.7540	0.4230	0.1370	0.039*	
C12	0.3992 (5)	0.4089 (4)	0.3878 (3)	0.0345 (14)	
H12A	0.4626	0.4022	0.3784	0.041*	
H12B	0.3841	0.4639	0.3847	0.041*	
C13	1.0959 (4)	0.2742 (4)	0.2288 (3)	0.0364 (15)	
H13A	1.1494	0.2968	0.2462	0.044*	
H13B	1.1057	0.2723	0.1900	0.044*	
C14	0.4142 (4)	0.2886 (4)	0.5899 (2)	0.0338 (14)	
H14A	0.3486	0.2900	0.5935	0.041*	
H14B	0.4412	0.3191	0.6192	0.041*	
C15	0.4357 (4)	0.4255 (4)	0.4782 (2)	0.0309 (13)	
H15A	0.4215	0.4805	0.4745	0.037*	
H15B	0.5000	0.4183	0.4716	0.037*	
C16	0.8531 (4)	0.2836 (4)	0.6720 (2)	0.0339 (15)	
H16A	0.8039	0.3134	0.6876	0.041*	
H16B	0.9029	0.2814	0.6983	0.041*	
C17	0.3543 (4)	0.1593 (4)	0.3171 (2)	0.0342 (15)	
H17A	0.3222	0.1282	0.2900	0.041*	
H17B	0.4180	0.1618	0.3076	0.041*	
C18	0.3778 (5)	0.0499 (4)	0.3712 (3)	0.0361 (15)	
H18A	0.4431	0.0494	0.3662	0.043*	
H18B	0.3495	0.0184	0.3429	0.043*	
C19	0.3411 (5)	0.3630 (4)	0.3518 (3)	0.0399 (16)	
H19A	0.2786	0.3664	0.3635	0.048*	
H19B	0.3441	0.3836	0.3152	0.048*	
C20	0.9467 (5)	0.4410 (4)	0.2360 (3)	0.0373 (15)	
H20A	0.9324	0.4347	0.2740	0.045*	
H20B	0.9545	0.4962	0.2287	0.045*	
C21	1.0715 (5)	0.4229 (4)	0.5247 (3)	0.0350 (15)	
H21A	1.0862	0.4766	0.5336	0.042*	
H21B	1.0336	0.4227	0.4920	0.042*	
C22	1.1822 (4)	0.1807 (4)	0.4696 (3)	0.0364 (15)	
H22A	1.1397	0.1833	0.4392	0.044*	
H22B	1.2345	0.1503	0.4587	0.044*	
C23	1.1561 (5)	0.3783 (4)	0.5152 (3)	0.0356 (15)	
H23A	1.1935	0.4057	0.4893	0.043*	
H23B	1.1906	0.3731	0.5489	0.043*	
C24	0.4307 (4)	0.0847 (4)	0.5562 (3)	0.0325 (14)	
H24A	0.4230	0.0635	0.5924	0.039*	
H24B	0.4950	0.0828	0.5480	0.039*	
C25	0.6518 (4)	0.3095 (4)	0.1461 (2)	0.0309 (14)	
H25A	0.6835	0.3085	0.1119	0.037*	
H25B	0.5963	0.3394	0.1411	0.037*	
C26	0.6299 (4)	0.2296 (4)	0.1628 (2)	0.0290 (13)	
H26A	0.6088	0.2294	0.1999	0.035*	
H26B	0.5819	0.2085	0.1396	0.035*	
C27	0.3799 (4)	0.0374 (4)	0.5172 (2)	0.0326 (14)	
H27A	0.3947	−0.0173	0.5224	0.039*	
H27B	0.3151	0.0441	0.5225	0.039*	
C28	0.4461 (5)	0.2083 (4)	0.5933 (2)	0.0349 (15)	
H28A	0.5110	0.2067	0.5869	0.042*	
H28B	0.4356	0.1879	0.6294	0.042*	
C29	0.9235 (5)	0.0484 (4)	0.2023 (3)	0.0363 (15)	
H29A	0.9462	0.0478	0.1656	0.044*	
H29B	0.9127	−0.0050	0.2134	0.044*	
C30	1.0316 (4)	0.3978 (4)	0.2233 (3)	0.0359 (15)	
H30A	1.0430	0.4002	0.1847	0.043*	
H30B	1.0831	0.4207	0.2426	0.043*	
C31	0.7950 (4)	0.4512 (4)	0.2124 (3)	0.0329 (14)	
H31A	0.8034	0.5069	0.2073	0.039*	
H31B	0.7742	0.4423	0.2491	0.039*	
C32	0.4138 (5)	0.3982 (4)	0.5338 (3)	0.0335 (14)	
H32A	0.4464	0.4296	0.5605	0.040*	
H32B	0.3491	0.4038	0.5399	0.040*	
C33	0.9898 (5)	0.0847 (4)	0.2385 (3)	0.0371 (15)	
H33A	0.9648	0.0893	0.2746	0.045*	
H33B	1.0440	0.0526	0.2410	0.045*	
C34	0.9447 (5)	0.4290 (4)	0.5816 (3)	0.0415 (17)	
H34A	0.8998	0.4242	0.5525	0.050*	
H34B	0.9588	0.4840	0.5862	0.050*	
C35	1.1112 (4)	0.0688 (4)	0.5032 (3)	0.0344 (15)	
H35A	1.1619	0.0381	0.4904	0.041*	
H35B	1.0645	0.0698	0.4749	0.041*	
C36	0.9074 (5)	0.3980 (4)	0.6319 (3)	0.0436 (17)	
H36A	0.9526	0.4016	0.6609	0.052*	
H36B	0.8547	0.4284	0.6422	0.052*	

### Atomic displacement parameters (Å^2^)

**Table:**

	*U*^11^	*U*^22^	*U*^33^	*U*^12^	*U*^13^	*U*^23^
Sb1	0.02325 (18)	0.0364 (2)	0.02034 (18)	0.00195 (16)	0.00063 (14)	0.00149 (16)
Sb2	0.0290 (2)	0.0282 (2)	0.02750 (19)	0.00258 (17)	−0.00079 (16)	0.00732 (17)
Sb3	0.0287 (2)	0.0238 (2)	0.02746 (19)	0.00118 (16)	0.00176 (15)	0.00238 (16)
Sb4	0.02578 (19)	0.0377 (2)	0.01891 (18)	−0.00189 (17)	0.00039 (14)	0.00531 (16)
Sb5	0.0313 (2)	0.0302 (2)	0.0263 (2)	−0.00022 (17)	0.00243 (16)	−0.00597 (17)
Sb6	0.02441 (18)	0.0355 (2)	0.02000 (18)	−0.00325 (16)	−0.00027 (14)	−0.00186 (16)
Sb7	0.0291 (2)	0.0292 (2)	0.0282 (2)	−0.00651 (17)	0.00153 (16)	0.00162 (17)
Rb1	0.0266 (3)	0.0286 (3)	0.0204 (2)	−0.0007 (2)	0.0009 (2)	0.0003 (2)
Rb2	0.0269 (3)	0.0317 (3)	0.0211 (2)	0.0005 (2)	0.0005 (2)	−0.0007 (2)
Rb3	0.0259 (3)	0.0281 (3)	0.0207 (2)	−0.0008 (2)	−0.0003 (2)	0.0015 (2)
O1	0.032 (2)	0.033 (2)	0.026 (2)	−0.0004 (19)	−0.0033 (18)	0.0000 (18)
O2	0.034 (2)	0.038 (3)	0.024 (2)	0.0038 (19)	0.0034 (18)	−0.0030 (18)
O3	0.039 (2)	0.031 (2)	0.027 (2)	−0.006 (2)	0.0001 (18)	0.0015 (18)
O4	0.031 (2)	0.034 (2)	0.027 (2)	0.0041 (19)	−0.0035 (18)	−0.0042 (18)
O5	0.041 (2)	0.029 (2)	0.030 (2)	0.0026 (19)	−0.0087 (19)	0.0010 (18)
O6	0.037 (2)	0.028 (2)	0.029 (2)	−0.0024 (19)	0.0029 (19)	0.0036 (18)
O7	0.029 (2)	0.037 (2)	0.0228 (19)	0.0026 (19)	0.0019 (17)	−0.0038 (18)
O8	0.026 (2)	0.033 (2)	0.026 (2)	−0.0021 (18)	0.0019 (17)	−0.0015 (18)
O9	0.028 (2)	0.039 (3)	0.034 (2)	−0.0061 (19)	−0.0028 (18)	−0.004 (2)
O10	0.044 (3)	0.034 (2)	0.023 (2)	0.001 (2)	0.0069 (18)	0.0001 (18)
O11	0.029 (2)	0.030 (2)	0.026 (2)	0.0036 (18)	0.0008 (17)	0.0003 (18)
O12	0.036 (2)	0.036 (3)	0.031 (2)	0.005 (2)	0.0000 (19)	0.0067 (19)
O13	0.028 (2)	0.039 (3)	0.033 (2)	−0.0030 (19)	−0.0010 (18)	0.007 (2)
O14	0.032 (2)	0.025 (2)	0.027 (2)	−0.0009 (17)	0.0009 (17)	0.0025 (17)
O15	0.037 (2)	0.027 (2)	0.023 (2)	−0.0008 (18)	−0.0034 (18)	0.0044 (17)
O16	0.034 (2)	0.038 (3)	0.033 (2)	0.003 (2)	−0.0037 (19)	0.002 (2)
O17	0.037 (2)	0.029 (2)	0.026 (2)	−0.0023 (19)	−0.0006 (18)	−0.0043 (18)
O18	0.036 (2)	0.024 (2)	0.035 (2)	0.0037 (18)	−0.0032 (19)	−0.0040 (19)
N1	0.065 (4)	0.038 (3)	0.038 (3)	−0.005 (3)	−0.006 (3)	0.002 (3)
N2	0.069 (4)	0.050 (4)	0.032 (3)	0.002 (3)	0.005 (3)	0.002 (3)
N3	0.039 (4)	0.101 (7)	0.064 (5)	−0.014 (4)	0.003 (3)	−0.023 (5)
N4	0.090 (6)	0.063 (5)	0.056 (4)	0.021 (4)	−0.025 (4)	0.006 (4)
C1	0.028 (3)	0.028 (3)	0.040 (3)	−0.001 (3)	0.002 (3)	−0.005 (3)
C2	0.040 (3)	0.030 (3)	0.025 (3)	−0.004 (3)	−0.005 (3)	−0.002 (3)
C3	0.040 (3)	0.027 (3)	0.022 (3)	−0.003 (3)	0.003 (3)	−0.001 (2)
C4	0.027 (3)	0.047 (4)	0.029 (3)	0.005 (3)	−0.003 (2)	−0.001 (3)
C5	0.033 (3)	0.026 (3)	0.029 (3)	−0.006 (3)	0.002 (3)	−0.003 (3)
C6	0.031 (3)	0.048 (4)	0.020 (3)	0.003 (3)	0.003 (2)	0.000 (3)
C7	0.044 (4)	0.047 (4)	0.020 (3)	0.001 (3)	−0.011 (3)	0.001 (3)
C8	0.025 (3)	0.026 (3)	0.039 (3)	0.005 (2)	−0.007 (3)	−0.010 (3)
C9	0.033 (3)	0.039 (4)	0.023 (3)	−0.010 (3)	0.004 (2)	0.003 (3)
C10	0.023 (3)	0.041 (4)	0.037 (3)	0.001 (3)	0.006 (3)	0.007 (3)
C11	0.037 (3)	0.029 (3)	0.030 (3)	0.002 (3)	−0.002 (3)	0.003 (3)
C12	0.044 (4)	0.024 (3)	0.035 (3)	0.001 (3)	0.001 (3)	0.008 (3)
C13	0.027 (3)	0.045 (4)	0.037 (3)	0.001 (3)	−0.001 (3)	−0.003 (3)
C14	0.037 (3)	0.043 (4)	0.022 (3)	−0.002 (3)	0.001 (3)	−0.007 (3)
C15	0.035 (3)	0.026 (3)	0.032 (3)	−0.004 (3)	0.005 (3)	−0.004 (3)
C16	0.031 (3)	0.045 (4)	0.025 (3)	0.008 (3)	0.005 (3)	−0.001 (3)
C17	0.034 (3)	0.044 (4)	0.024 (3)	−0.003 (3)	−0.007 (3)	−0.006 (3)
C18	0.033 (3)	0.038 (4)	0.037 (3)	0.004 (3)	−0.004 (3)	−0.012 (3)
C19	0.045 (4)	0.043 (4)	0.032 (3)	0.012 (3)	−0.006 (3)	0.006 (3)
C20	0.040 (4)	0.039 (4)	0.032 (3)	−0.012 (3)	−0.005 (3)	−0.001 (3)
C21	0.044 (4)	0.029 (3)	0.033 (3)	−0.002 (3)	−0.003 (3)	0.006 (3)
C22	0.027 (3)	0.050 (4)	0.032 (3)	0.001 (3)	0.003 (3)	−0.006 (3)
C23	0.038 (3)	0.035 (4)	0.033 (3)	−0.006 (3)	0.003 (3)	0.005 (3)
C24	0.038 (3)	0.029 (3)	0.031 (3)	0.005 (3)	0.007 (3)	0.009 (3)
C25	0.034 (3)	0.033 (3)	0.026 (3)	−0.001 (3)	−0.007 (3)	−0.002 (3)
C26	0.028 (3)	0.033 (3)	0.026 (3)	0.000 (3)	−0.005 (2)	0.001 (3)
C27	0.035 (3)	0.033 (3)	0.031 (3)	−0.001 (3)	0.008 (3)	0.002 (3)
C28	0.039 (3)	0.044 (4)	0.022 (3)	−0.001 (3)	0.003 (3)	−0.002 (3)
C29	0.043 (4)	0.029 (3)	0.037 (3)	0.008 (3)	0.000 (3)	0.002 (3)
C30	0.031 (3)	0.038 (4)	0.038 (3)	−0.015 (3)	−0.002 (3)	0.003 (3)
C31	0.041 (4)	0.025 (3)	0.032 (3)	0.002 (3)	0.002 (3)	0.000 (3)
C32	0.035 (3)	0.036 (4)	0.030 (3)	−0.008 (3)	0.004 (3)	−0.007 (3)
C33	0.043 (4)	0.031 (4)	0.037 (3)	0.006 (3)	−0.001 (3)	0.003 (3)
C34	0.043 (4)	0.030 (4)	0.052 (4)	0.016 (3)	0.009 (3)	0.006 (3)
C35	0.025 (3)	0.035 (4)	0.043 (4)	0.002 (3)	0.001 (3)	−0.017 (3)
C36	0.055 (4)	0.036 (4)	0.041 (4)	0.012 (3)	0.015 (3)	−0.004 (3)

### Geometric parameters (Å, °)

**Table:**

Sb1—Sb7	2.7647 (8)	N4—H4D	0.9100
Sb1—Sb3	2.8090 (8)	N4—H4E	0.9100
Sb1—Rb2	3.7411 (10)	C1—C18	1.502 (10)
Sb1—Rb3	3.8327 (11)	C1—H1A	0.9900
Sb2—Sb6	2.7477 (8)	C1—H1B	0.9900
Sb2—Sb5	2.8687 (8)	C2—C9	1.509 (10)
Sb2—Sb7	2.8790 (9)	C2—H2A	0.9900
Sb2—Rb3	4.2005 (13)	C2—H2B	0.9900
Sb2—Rb1	4.2703 (12)	C3—C5	1.518 (9)
Sb3—Sb6	2.7544 (9)	C3—H3A	0.9900
Sb3—Sb4	2.8215 (8)	C3—H3B	0.9900
Sb4—Sb5	2.7587 (8)	C4—C13	1.503 (10)
Sb4—Rb1	3.8756 (12)	C4—H4A	0.9900
Sb4—Rb2	3.9161 (11)	C4—H4B	0.9900
Sb5—Sb7	2.9298 (10)	C5—H5A	0.9900
Sb5—Rb1	4.3283 (10)	C5—H5B	0.9900
Sb6—Rb1	3.8465 (13)	C6—C16	1.492 (10)
Sb6—Rb3	3.8862 (13)	C6—H6A	0.9900
Sb7—Rb3	4.1688 (10)	C6—H6B	0.9900
Sb7—Rb2	4.3337 (11)	C7—C17	1.490 (10)
Rb1—O2	2.963 (4)	C7—H7A	0.9900
Rb1—O6	2.982 (4)	C7—H7B	0.9900
Rb1—O5	2.988 (4)	C8—C35	1.505 (10)
Rb1—O1	3.100 (4)	C8—H8A	0.9900
Rb1—O3	3.103 (5)	C8—H8B	0.9900
Rb1—O4	3.115 (4)	C9—H9A	0.9900
Rb1—C24	3.690 (6)	C9—H9B	0.9900
Rb1—C15	3.717 (7)	C10—C22	1.488 (10)
Rb1—C28	3.728 (6)	C10—H10A	0.9900
Rb2—O11	2.993 (4)	C10—H10B	0.9900
Rb2—O10	3.021 (5)	C11—C31	1.488 (9)
Rb2—O12	3.056 (5)	C11—H11A	0.9900
Rb2—O9	3.092 (5)	C11—H11B	0.9900
Rb2—O8	3.139 (4)	C12—C19	1.483 (10)
Rb2—O7	3.148 (4)	C12—H12A	0.9900
Rb2—C34	3.696 (8)	C12—H12B	0.9900
Rb2—C35	3.708 (7)	C13—H13A	0.9900
Rb3—O18	2.934 (4)	C13—H13B	0.9900
Rb3—O15	2.956 (4)	C14—C28	1.485 (10)
Rb3—O13	3.047 (4)	C14—H14A	0.9900
Rb3—O14	3.082 (4)	C14—H14B	0.9900
Rb3—O17	3.119 (5)	C15—C32	1.518 (9)
Rb3—O16	3.160 (5)	C15—H15A	0.9900
Rb3—C33	3.679 (7)	C15—H15B	0.9900
Rb3—C5	3.696 (6)	C16—H16A	0.9900
Rb3—C26	3.704 (6)	C16—H16B	0.9900
Rb3—C31	3.705 (7)	C17—H17A	0.9900
O1—C28	1.421 (8)	C17—H17B	0.9900
O1—C24	1.426 (8)	C18—H18A	0.9900
O2—C32	1.413 (8)	C18—H18B	0.9900
O2—C14	1.455 (8)	C19—H19A	0.9900
O3—C15	1.407 (7)	C19—H19B	0.9900
O3—C12	1.446 (8)	C20—C30	1.519 (10)
O4—C18	1.421 (8)	C20—H20A	0.9900
O4—C17	1.437 (8)	C20—H20B	0.9900
O5—C7	1.413 (8)	C21—C23	1.513 (10)
O5—C19	1.421 (8)	C21—H21A	0.9900
O6—C1	1.414 (7)	C21—H21B	0.9900
O6—C27	1.438 (8)	C22—H22A	0.9900
O7—C9	1.429 (8)	C22—H22B	0.9900
O7—C6	1.448 (8)	C23—H23A	0.9900
O8—C35	1.420 (8)	C23—H23B	0.9900
O8—C22	1.450 (8)	C24—C27	1.483 (9)
O9—C23	1.420 (8)	C24—H24A	0.9900
O9—C10	1.433 (8)	C24—H24B	0.9900
O10—C16	1.433 (8)	C25—C26	1.497 (9)
O10—C36	1.435 (9)	C25—H25A	0.9900
O11—C8	1.432 (7)	C25—H25B	0.9900
O11—C2	1.432 (8)	C26—H26A	0.9900
O12—C34	1.435 (8)	C26—H26B	0.9900
O12—C21	1.440 (8)	C27—H27A	0.9900
O13—C13	1.426 (8)	C27—H27B	0.9900
O13—C30	1.437 (8)	C28—H28A	0.9900
O14—C26	1.432 (8)	C28—H28B	0.9900
O14—C5	1.432 (7)	C29—C33	1.481 (10)
O15—C25	1.422 (7)	C29—H29A	0.9900
O15—C11	1.422 (8)	C29—H29B	0.9900
O16—C33	1.423 (8)	C30—H30A	0.9900
O16—C4	1.426 (8)	C30—H30B	0.9900
O17—C20	1.401 (7)	C31—H31A	0.9900
O17—C31	1.442 (8)	C31—H31B	0.9900
O18—C3	1.406 (7)	C32—H32A	0.9900
O18—C29	1.426 (8)	C32—H32B	0.9900
N1—H1C	0.9100	C33—H33A	0.9900
N1—H1D	0.9100	C33—H33B	0.9900
N1—H1E	0.9100	C34—C36	1.496 (11)
N2—H2C	0.9100	C34—H34A	0.9900
N2—H2D	0.9100	C34—H34B	0.9900
N2—H2E	0.9100	C35—H35A	0.9900
N3—H3C	0.9100	C35—H35B	0.9900
N3—H3D	0.9100	C36—H36A	0.9900
N3—H3E	0.9100	C36—H36B	0.9900
N4—H4C	0.9100		
			
Sb7—Sb1—Sb3	98.58 (2)	C31—O17—Rb3	102.3 (3)
Sb7—Sb1—Rb2	82.056 (19)	C3—O18—C29	111.5 (5)
Sb3—Sb1—Rb2	89.37 (3)	C3—O18—Rb3	122.6 (4)
Sb7—Sb1—Rb3	76.480 (17)	C29—O18—Rb3	122.4 (4)
Sb3—Sb1—Rb3	92.14 (3)	C29^i^—N1—H1C	109.5
Rb2—Sb1—Rb3	158.469 (19)	C29^i^—N1—H1D	109.5
Sb6—Sb2—Sb5	106.300 (18)	H1C—N1—H1D	109.5
Sb6—Sb2—Sb7	105.516 (18)	C29^i^—N1—H1E	109.5
Sb5—Sb2—Sb7	61.29 (3)	H1C—N1—H1E	109.5
Sb6—Sb2—Rb3	64.078 (16)	H1D—N1—H1E	109.5
Sb5—Sb2—Rb3	124.67 (2)	C16—N2—H2C	109.5
Sb7—Sb2—Rb3	69.29 (2)	C16—N2—H2D	109.5
Sb6—Sb2—Rb1	62.076 (17)	H2C—N2—H2D	109.5
Sb5—Sb2—Rb1	71.61 (2)	C16—N2—H2E	109.5
Sb7—Sb2—Rb1	125.38 (2)	H2C—N2—H2E	109.5
Rb3—Sb2—Rb1	126.15 (2)	H2D—N2—H2E	109.5
Sb6—Sb3—Sb1	101.18 (2)	C19^ii^—N3—H3C	109.5
Sb6—Sb3—Sb4	101.67 (2)	C19^ii^—N3—H3D	109.5
Sb1—Sb3—Sb4	102.84 (3)	H3C—N3—H3D	109.5
Sb5—Sb4—Sb3	98.63 (2)	C19^ii^—N3—H3E	109.5
Sb5—Sb4—Rb1	79.500 (16)	H3C—N3—H3E	109.5
Sb3—Sb4—Rb1	89.56 (3)	H3D—N3—H3E	109.5
Sb5—Sb4—Rb2	81.462 (18)	C36—N4—H4C	109.5
Sb3—Sb4—Rb2	85.74 (3)	C36—N4—H4D	109.5
Rb1—Sb4—Rb2	159.462 (18)	H4C—N4—H4D	109.5
Sb4—Sb5—Sb2	104.337 (18)	C36—N4—H4E	109.5
Sb4—Sb5—Sb7	105.357 (19)	H4C—N4—H4E	109.5
Sb2—Sb5—Sb7	59.53 (2)	H4D—N4—H4E	109.5
Sb4—Sb5—Rb1	61.694 (18)	O6—C1—C18	108.9 (5)
Sb2—Sb5—Rb1	69.42 (3)	O6—C1—H1A	109.9
Sb7—Sb5—Rb1	122.01 (2)	C18—C1—H1A	109.9
Sb2—Sb6—Sb3	98.62 (2)	O6—C1—H1B	109.9
Sb2—Sb6—Rb1	78.789 (16)	C18—C1—H1B	109.9
Sb3—Sb6—Rb1	91.16 (2)	H1A—C1—H1B	108.3
Sb2—Sb6—Rb3	76.436 (17)	O11—C2—C9	110.5 (5)
Sb3—Sb6—Rb3	91.86 (2)	O11—C2—H2A	109.5
Rb1—Sb6—Rb3	155.215 (19)	C9—C2—H2A	109.5
Sb1—Sb7—Sb2	104.330 (18)	O11—C2—H2B	109.5
Sb1—Sb7—Sb5	105.546 (19)	C9—C2—H2B	109.5
Sb2—Sb7—Sb5	59.18 (2)	H2A—C2—H2B	108.1
Sb1—Sb7—Rb3	63.368 (18)	O18—C3—C5	108.0 (5)
Sb2—Sb7—Rb3	70.47 (3)	O18—C3—H3A	110.1
Sb5—Sb7—Rb3	123.89 (2)	C5—C3—H3A	110.1
Sb1—Sb7—Rb2	58.758 (15)	O18—C3—H3B	110.1
Sb2—Sb7—Rb2	122.20 (3)	C5—C3—H3B	110.1
Sb5—Sb7—Rb2	72.52 (2)	H3A—C3—H3B	108.4
Rb3—Sb7—Rb2	122.10 (2)	O16—C4—C13	109.4 (5)
O2—Rb1—O6	112.49 (13)	O16—C4—H4A	109.8
O2—Rb1—O5	108.67 (12)	C13—C4—H4A	109.8
O6—Rb1—O5	108.84 (12)	O16—C4—H4B	109.8
O2—Rb1—O1	56.25 (12)	C13—C4—H4B	109.8
O6—Rb1—O1	56.68 (12)	H4A—C4—H4B	108.3
O5—Rb1—O1	131.47 (12)	O14—C5—C3	107.7 (5)
O2—Rb1—O3	54.70 (12)	O14—C5—Rb3	54.1 (3)
O6—Rb1—O3	136.97 (12)	C3—C5—Rb3	85.2 (3)
O5—Rb1—O3	55.15 (12)	O14—C5—H5A	110.2
O1—Rb1—O3	101.42 (12)	C3—C5—H5A	110.2
O2—Rb1—O4	135.62 (12)	Rb3—C5—H5A	161.8
O6—Rb1—O4	54.27 (12)	O14—C5—H5B	110.2
O5—Rb1—O4	55.45 (12)	C3—C5—H5B	110.2
O1—Rb1—O4	100.68 (12)	Rb3—C5—H5B	73.4
O3—Rb1—O4	103.28 (11)	H5A—C5—H5B	108.5
O2—Rb1—C24	76.34 (14)	O7—C6—C16	109.0 (5)
O6—Rb1—C24	40.06 (13)	O7—C6—H6A	109.9
O5—Rb1—C24	139.61 (14)	C16—C6—H6A	109.9
O1—Rb1—C24	22.14 (13)	O7—C6—H6B	109.9
O3—Rb1—C24	123.46 (14)	C16—C6—H6B	109.9
O4—Rb1—C24	92.17 (14)	H6A—C6—H6B	108.3
O2—Rb1—C15	39.89 (13)	O5—C7—C17	110.4 (5)
O6—Rb1—C15	145.70 (14)	O5—C7—H7A	109.6
O5—Rb1—C15	74.84 (13)	C17—C7—H7A	109.6
O1—Rb1—C15	94.53 (13)	O5—C7—H7B	109.6
O3—Rb1—C15	21.48 (12)	C17—C7—H7B	109.6
O4—Rb1—C15	124.72 (13)	H7A—C7—H7B	108.1
C24—Rb1—C15	115.80 (15)	O11—C8—C35	111.5 (5)
O2—Rb1—C28	39.92 (14)	O11—C8—H8A	109.3
O6—Rb1—C28	76.32 (14)	C35—C8—H8A	109.3
O5—Rb1—C28	139.18 (14)	O11—C8—H8B	109.3
O1—Rb1—C28	21.61 (13)	C35—C8—H8B	109.3
O3—Rb1—C28	92.66 (14)	H8A—C8—H8B	108.0
O4—Rb1—C28	122.14 (14)	O7—C9—C2	108.3 (5)
C24—Rb1—C28	37.16 (15)	O7—C9—H9A	110.0
C15—Rb1—C28	79.81 (15)	C2—C9—H9A	110.0
O2—Rb1—Sb6	138.65 (9)	O7—C9—H9B	110.0
O6—Rb1—Sb6	101.61 (9)	C2—C9—H9B	110.0
O5—Rb1—Sb6	80.09 (10)	H9A—C9—H9B	108.4
O1—Rb1—Sb6	144.18 (8)	O9—C10—C22	109.0 (5)
O3—Rb1—Sb6	112.34 (9)	O9—C10—H10A	109.9
O4—Rb1—Sb6	83.35 (9)	C22—C10—H10A	109.9
C24—Rb1—Sb6	123.41 (11)	O9—C10—H10B	109.9
C15—Rb1—Sb6	112.50 (10)	C22—C10—H10B	109.9
C28—Rb1—Sb6	139.90 (11)	H10A—C10—H10B	108.3
O2—Rb1—Sb4	84.49 (9)	O15—C11—C31	109.9 (5)
O6—Rb1—Sb4	97.44 (8)	O15—C11—H11A	109.7
O5—Rb1—Sb4	142.20 (10)	C31—C11—H11A	109.7
O1—Rb1—Sb4	85.63 (8)	O15—C11—H11B	109.7
O3—Rb1—Sb4	118.98 (8)	C31—C11—H11B	109.7
O4—Rb1—Sb4	135.22 (8)	H11A—C11—H11B	108.2
C24—Rb1—Sb4	77.24 (11)	O3—C12—C19	107.5 (6)
C15—Rb1—Sb4	98.44 (10)	O3—C12—H12A	110.2
C28—Rb1—Sb4	72.46 (11)	C19—C12—H12A	110.2
Sb6—Rb1—Sb4	68.09 (2)	O3—C12—H12B	110.2
O2—Rb1—Sb2	102.02 (9)	C19—C12—H12B	110.2
O6—Rb1—Sb2	140.18 (9)	H12A—C12—H12B	108.5
O5—Rb1—Sb2	76.56 (10)	O13—C13—C4	108.9 (6)
O1—Rb1—Sb2	146.73 (8)	O13—C13—H13A	109.9
O3—Rb1—Sb2	79.50 (9)	C4—C13—H13A	109.9
O4—Rb1—Sb2	111.56 (9)	O13—C13—H13B	109.9
C24—Rb1—Sb2	143.06 (11)	C4—C13—H13B	109.9
C15—Rb1—Sb2	74.11 (10)	H13A—C13—H13B	108.3
C28—Rb1—Sb2	126.03 (11)	O2—C14—C28	109.1 (5)
Sb6—Rb1—Sb2	39.136 (16)	O2—C14—H14A	109.9
Sb4—Rb1—Sb2	65.936 (18)	C28—C14—H14A	109.9
O11—Rb2—O10	109.50 (12)	O2—C14—H14B	109.9
O11—Rb2—O12	136.21 (11)	C28—C14—H14B	109.9
O10—Rb2—O12	55.84 (13)	H14A—C14—H14B	108.3
O11—Rb2—O9	110.55 (12)	O3—C15—C32	108.3 (5)
O10—Rb2—O9	110.66 (13)	O3—C15—Rb1	53.9 (3)
O12—Rb2—O9	55.83 (12)	C32—C15—Rb1	85.8 (4)
O11—Rb2—O8	56.94 (12)	O3—C15—H15A	110.0
O10—Rb2—O8	134.67 (12)	C32—C15—H15A	110.0
O12—Rb2—O8	101.66 (12)	Rb1—C15—H15A	161.4
O9—Rb2—O8	54.31 (12)	O3—C15—H15B	110.0
O11—Rb2—O7	55.35 (12)	C32—C15—H15B	110.0
O10—Rb2—O7	54.71 (12)	Rb1—C15—H15B	73.4
O12—Rb2—O7	101.80 (12)	H15A—C15—H15B	108.4
O9—Rb2—O7	134.33 (11)	O10—C16—C6	108.7 (5)
O8—Rb2—O7	102.35 (11)	O10—C16—N2	76.7 (3)
O11—Rb2—C34	143.50 (14)	C6—C16—N2	96.0 (4)
O10—Rb2—C34	40.21 (15)	O10—C16—H16A	109.9
O12—Rb2—C34	22.03 (15)	C6—C16—H16A	109.9
O9—Rb2—C34	76.05 (15)	O10—C16—H16B	109.9
O8—Rb2—C34	123.60 (14)	C6—C16—H16B	109.9
O7—Rb2—C34	93.55 (14)	H16A—C16—H16B	108.3
O11—Rb2—C35	40.75 (14)	O4—C17—C7	107.1 (5)
O10—Rb2—C35	142.42 (14)	O4—C17—H17A	110.3
O12—Rb2—C35	123.54 (13)	C7—C17—H17A	110.3
O9—Rb2—C35	74.45 (14)	O4—C17—H17B	110.3
O8—Rb2—C35	21.98 (13)	C7—C17—H17B	110.3
O7—Rb2—C35	94.15 (14)	H17A—C17—H17B	108.5
C34—Rb2—C35	145.32 (15)	O4—C18—C1	107.3 (5)
O11—Rb2—Sb1	100.66 (8)	O4—C18—H18A	110.3
O10—Rb2—Sb1	138.52 (9)	C1—C18—H18A	110.3
O12—Rb2—Sb1	116.45 (8)	O4—C18—H18B	110.3
O9—Rb2—Sb1	83.27 (8)	C1—C18—H18B	110.3
O8—Rb2—Sb1	85.69 (8)	H18A—C18—H18B	108.5
O7—Rb2—Sb1	138.56 (8)	O5—C19—C12	110.1 (5)
C34—Rb2—Sb1	115.84 (12)	O5—C19—H19A	109.6
C35—Rb2—Sb1	78.27 (11)	C12—C19—H19A	109.6
O11—Rb2—Sb4	93.18 (8)	O5—C19—H19B	109.6
O10—Rb2—Sb4	80.19 (9)	C12—C19—H19B	109.6
O12—Rb2—Sb4	119.93 (9)	H19A—C19—H19B	108.2
O9—Rb2—Sb4	147.33 (8)	O17—C20—C30	108.0 (6)
O8—Rb2—Sb4	137.74 (8)	O17—C20—H20A	110.1
O7—Rb2—Sb4	77.55 (8)	C30—C20—H20A	110.1
C34—Rb2—Sb4	98.36 (12)	O17—C20—H20B	110.1
C35—Rb2—Sb4	116.32 (10)	C30—C20—H20B	110.1
Sb1—Rb2—Sb4	70.13 (3)	H20A—C20—H20B	108.4
O11—Rb2—Sb7	138.25 (8)	O12—C21—C23	109.2 (5)
O10—Rb2—Sb7	102.51 (9)	O12—C21—H21A	109.8
O12—Rb2—Sb7	84.18 (8)	C23—C21—H21A	109.8
O9—Rb2—Sb7	81.13 (8)	O12—C21—H21B	109.8
O8—Rb2—Sb7	114.54 (8)	C23—C21—H21B	109.8
O7—Rb2—Sb7	140.69 (8)	H21A—C21—H21B	108.3
C34—Rb2—Sb7	77.55 (12)	O8—C22—C10	109.3 (5)
C35—Rb2—Sb7	114.99 (11)	O8—C22—H22A	109.8
Sb1—Rb2—Sb7	39.186 (15)	C10—C22—H22A	109.8
Sb4—Rb2—Sb7	66.30 (2)	O8—C22—H22B	109.8
O18—Rb3—O15	112.22 (12)	C10—C22—H22B	109.8
O18—Rb3—O13	109.16 (13)	H22A—C22—H22B	108.3
O15—Rb3—O13	108.75 (12)	O9—C23—C21	109.2 (5)
O18—Rb3—O14	55.56 (11)	O9—C23—H23A	109.8
O15—Rb3—O14	57.05 (11)	C21—C23—H23A	109.8
O13—Rb3—O14	131.45 (12)	O9—C23—H23B	109.8
O18—Rb3—O17	136.49 (12)	C21—C23—H23B	109.8
O15—Rb3—O17	55.88 (11)	H23A—C23—H23B	108.3
O13—Rb3—O17	53.93 (12)	O1—C24—C27	110.9 (5)
O14—Rb3—O17	102.66 (11)	O1—C24—Rb1	55.0 (3)
O18—Rb3—O16	55.05 (12)	C27—C24—Rb1	86.6 (4)
O15—Rb3—O16	135.67 (12)	O1—C24—H24A	109.5
O13—Rb3—O16	55.02 (13)	C27—C24—H24A	109.5
O14—Rb3—O16	100.35 (12)	Rb1—C24—H24A	161.8
O17—Rb3—O16	101.68 (12)	O1—C24—H24B	109.5
O18—Rb3—C33	39.90 (14)	C27—C24—H24B	109.5
O15—Rb3—C33	145.38 (14)	Rb1—C24—H24B	72.9
O13—Rb3—C33	75.20 (15)	H24A—C24—H24B	108.1
O14—Rb3—C33	93.78 (13)	O15—C25—C26	109.1 (5)
O17—Rb3—C33	124.09 (15)	O15—C25—H25A	109.9
O16—Rb3—C33	22.41 (15)	C26—C25—H25A	109.9
O18—Rb3—C5	39.78 (13)	O15—C25—H25B	109.9
O15—Rb3—C5	76.65 (13)	C26—C25—H25B	109.9
O13—Rb3—C5	140.34 (14)	H25A—C25—H25B	108.3
O14—Rb3—C5	22.10 (12)	O14—C26—C25	108.4 (5)
O17—Rb3—C5	124.71 (12)	O14—C26—Rb3	53.7 (2)
O16—Rb3—C5	92.90 (13)	C25—C26—Rb3	83.9 (3)
C33—Rb3—C5	79.66 (15)	O14—C26—H26A	110.0
O18—Rb3—C26	75.80 (13)	C25—C26—H26A	110.0
O15—Rb3—C26	39.89 (13)	Rb3—C26—H26A	75.2
O13—Rb3—C26	138.83 (13)	O14—C26—H26B	110.0
O14—Rb3—C26	22.00 (13)	C25—C26—H26B	110.0
O17—Rb3—C26	93.47 (13)	Rb3—C26—H26B	162.3
O16—Rb3—C26	122.08 (13)	H26A—C26—H26B	108.4
C33—Rb3—C26	115.15 (15)	O6—C27—C24	109.1 (5)
C5—Rb3—C26	37.26 (14)	O6—C27—H27A	109.9
O18—Rb3—C31	145.24 (13)	C24—C27—H27A	109.9
O15—Rb3—C31	39.95 (13)	O6—C27—H27B	109.9
O13—Rb3—C31	74.57 (14)	C24—C27—H27B	109.9
O14—Rb3—C31	95.20 (12)	H27A—C27—H27B	108.3
O17—Rb3—C31	22.34 (14)	O1—C28—C14	110.4 (5)
O16—Rb3—C31	123.96 (14)	O1—C28—Rb1	53.5 (3)
C33—Rb3—C31	146.35 (16)	C14—C28—Rb1	85.7 (4)
C5—Rb3—C31	116.17 (14)	O1—C28—H28A	109.6
C26—Rb3—C31	79.84 (14)	C14—C28—H28A	109.6
O18—Rb3—Sb1	97.27 (8)	Rb1—C28—H28A	75.0
O15—Rb3—Sb1	142.00 (9)	O1—C28—H28B	109.6
O13—Rb3—Sb1	81.83 (8)	C14—C28—H28B	109.6
O14—Rb3—Sb1	140.25 (8)	Rb1—C28—H28B	161.4
O17—Rb3—Sb1	116.13 (7)	H28A—C28—H28B	108.1
O16—Rb3—Sb1	80.79 (8)	O18—C29—C33	108.7 (6)
C33—Rb3—Sb1	72.17 (11)	O18—C29—H29A	110.0
C5—Rb3—Sb1	118.78 (10)	C33—C29—H29A	110.0
C26—Rb3—Sb1	139.12 (10)	O18—C29—H29B	110.0
C31—Rb3—Sb1	117.30 (10)	C33—C29—H29B	110.0
O18—Rb3—Sb6	89.94 (10)	H29A—C29—H29B	108.3
O15—Rb3—Sb6	88.23 (9)	O13—C30—C20	107.6 (5)
O13—Rb3—Sb6	146.00 (9)	O13—C30—H30A	110.2
O14—Rb3—Sb6	82.54 (8)	C20—C30—H30A	110.2
O17—Rb3—Sb6	127.22 (9)	O13—C30—H30B	110.2
O16—Rb3—Sb6	129.42 (9)	C20—C30—H30B	110.2
C33—Rb3—Sb6	107.56 (12)	H30A—C30—H30B	108.5
C5—Rb3—Sb6	71.16 (10)	O17—C31—C11	107.2 (5)
C26—Rb3—Sb6	72.02 (10)	O17—C31—Rb3	55.3 (3)
C31—Rb3—Sb6	105.73 (11)	C11—C31—Rb3	85.0 (4)
Sb1—Rb3—Sb6	67.68 (2)	O17—C31—H31A	110.3
C28—O1—C24	112.2 (5)	C11—C31—H31A	110.3
C28—O1—Rb1	104.9 (4)	Rb3—C31—H31A	162.6
C24—O1—Rb1	102.9 (3)	O17—C31—H31B	110.3
C32—O2—C14	112.3 (5)	C11—C31—H31B	110.3
C32—O2—Rb1	122.6 (3)	Rb3—C31—H31B	72.0
C14—O2—Rb1	120.5 (4)	H31A—C31—H31B	108.5
C15—O3—C12	110.7 (5)	O2—C32—C15	108.9 (5)
C15—O3—Rb1	104.6 (4)	O2—C32—H32A	109.9
C12—O3—Rb1	105.2 (4)	C15—C32—H32A	109.9
C18—O4—C17	112.2 (5)	O2—C32—H32B	109.9
C18—O4—Rb1	105.5 (3)	C15—C32—H32B	109.9
C17—O4—Rb1	106.6 (3)	H32A—C32—H32B	108.3
C7—O5—C19	113.3 (5)	O16—C33—C29	109.6 (5)
C7—O5—Rb1	120.2 (4)	O16—C33—Rb3	57.8 (3)
C19—O5—Rb1	120.9 (3)	C29—C33—Rb3	86.8 (4)
C1—O6—C27	112.0 (5)	O16—C33—H33A	109.8
C1—O6—Rb1	122.6 (4)	C29—C33—H33A	109.8
C27—O6—Rb1	119.3 (4)	Rb3—C33—H33A	69.1
C9—O7—C6	112.2 (5)	O16—C33—H33B	109.8
C9—O7—Rb2	105.5 (3)	C29—C33—H33B	109.8
C6—O7—Rb2	107.2 (3)	Rb3—C33—H33B	162.6
C35—O8—C22	112.7 (5)	H33A—C33—H33B	108.2
C35—O8—Rb2	102.2 (3)	O12—C34—C36	110.4 (6)
C22—O8—Rb2	107.3 (3)	O12—C34—Rb2	53.0 (3)
C23—O9—C10	112.2 (5)	C36—C34—Rb2	87.8 (4)
C23—O9—Rb2	118.0 (4)	O12—C34—H34A	109.6
C10—O9—Rb2	120.9 (4)	C36—C34—H34A	109.6
C16—O10—C36	112.9 (5)	Rb2—C34—H34A	73.7
C16—O10—Rb2	122.4 (4)	O12—C34—H34B	109.6
C36—O10—Rb2	119.6 (4)	C36—C34—H34B	109.6
C8—O11—C2	114.3 (5)	Rb2—C34—H34B	159.9
C8—O11—Rb2	118.0 (4)	H34A—C34—H34B	108.1
C2—O11—Rb2	121.2 (4)	O8—C35—C8	109.6 (5)
C34—O12—C21	113.3 (5)	O8—C35—Rb2	55.8 (3)
C34—O12—Rb2	104.9 (4)	C8—C35—Rb2	85.0 (4)
C21—O12—Rb2	108.9 (4)	O8—C35—H35A	109.7
C13—O13—C30	111.8 (5)	C8—C35—H35A	109.7
C13—O13—Rb3	120.7 (4)	Rb2—C35—H35A	163.1
C30—O13—Rb3	121.8 (3)	O8—C35—H35B	109.7
C26—O14—C5	111.3 (5)	C8—C35—H35B	109.7
C26—O14—Rb3	104.3 (3)	Rb2—C35—H35B	72.8
C5—O14—Rb3	103.8 (3)	H35A—C35—H35B	108.2
C25—O15—C11	112.1 (4)	O10—C36—C34	109.5 (6)
C25—O15—Rb3	118.7 (4)	O10—C36—N4	107.7 (4)
C11—O15—Rb3	120.2 (3)	C34—C36—N4	129.6 (5)
C33—O16—C4	112.8 (5)	O10—C36—H36A	109.8
C33—O16—Rb3	99.8 (4)	C34—C36—H36A	109.8
C4—O16—Rb3	105.5 (4)	O10—C36—H36B	109.8
C20—O17—C31	111.4 (5)	C34—C36—H36B	109.8
C20—O17—Rb3	107.5 (4)	H36A—C36—H36B	108.2

Symmetry codes: (i) −*x*+3/2, *y*+1/2, −*z*+1/2; (ii) *x*+1, *y*, *z*.
